# The Evaluation of Skin Turgor in Relation to Changes in Intracranial Pressure in Patients After Decompressive Hemicraniectomy

**DOI:** 10.7759/cureus.29828

**Published:** 2022-10-02

**Authors:** Andrew Ku, Imran Siddiqi, Shivum Desai, Arman Saied, Dan E Miulli

**Affiliations:** 1 Neurosurgery, California University of Science and Medicine, Colton, USA; 2 Neurosurgery, Riverside University Health System Medical Center, Moreno Valley, USA; 3 Neurosurgery, Midwestern University Arizona College of Osteopathic Medicine, Glendale, USA; 4 Neurosurgery, Arrowhead Regional Medical Center, Colton, USA

**Keywords:** elevated icp, external ventricular drain, hemicraniectomy, decompressive craniectomy, intracranial pressure

## Abstract

Introduction

Decompressive hemicraniectomies have been the mainstay of treating medically refractory elevated intracranial pressures (ICPs). Afterward, ICP continues to be monitored. However, the reliability of monitoring the ICP in a patient after craniectomy has been shown to be variable, at best. We propose the use of a durometer to investigate a temporal relationship between skin turgor and elevated ICP.

Methods

Patients were included via the following criteria: age >18 and unilateral decompressive craniectomy, with an external ventricular drain (EVD) in place. Patients were excluded if they were younger than 18, underwent bilateral decompressive craniectomy, or did not have an ICP monitor. Skin turgor over the skin flap was measured with a durometer over the center of the defect. ICPs were monitored using an EVD. The optic nerve sheath diameter (ONSD) was measured with ultrasound with the eye closed and Tegaderm (3M, Saint Paul, MN) covering the eyelid. The optic nerve was measured 3 mm behind the globe, and the diameter of the optic nerve at the widest point was recorded. The Neurological Pupil index (NPi) was recorded with a pupillometer.

Results

Fourteen patients were included, with over 100 data points for ICP, skin turgor, ONSD, and NPi. Five patients went on to have elevated ICP after decompressive hemicraniectomy. The correlation coefficient (R) for ONSD to ICP correlation was 0.62. The R for ICP to skin turgor was 0.31. The data shows that a skin turgor of >9 is related to increasing ICP within 24 hours, a skin turgor of 6-9 is a warning, and a skin turgor of <6 is normal.

Conclusion

A temporal relationship between skin turgor and ICP exists, which could be used to predict impending elevations in ICP sooner than an ICP monitor can determine. By using this in conjunction with traditional methods of evaluating these patients, we could sooner act on elevations in ICP and potentially improve outcomes.

## Introduction

The purpose of a decompressive hemicraniectomy is to decrease intracranial pressure (ICP) [1], often performed for stroke [2] or traumatic brain injuries [3]. Intracranial pressure (ICP) monitoring is particularly valuable in cases where a craniectomy has been done specifically for decompression, as increased ICP is a warning sign of neurological deterioration for patients who have had decompressive craniectomies [4] for stroke [5,6] or traumatic causes [7-9].

After a craniectomy, the pressure within the head can be gauged by palpating the skin flap and feeling whether the skin is tense. The skin is more compliant than the bone, so the brain can swell into tissue to a greater degree than the bone, and there are proposed classification systems to assess skin flaps after craniectomies [10]. Palpation of the skin, however, is subjective, which can lead to more variable results than using an instrument.

External ventricular drain (EVD) measurement of ICP has theoretical limits for patients with craniectomies. ICP tends to be lower in patients who have had decompressive craniectomies because there is no bone, creating a larger reserve of space, so the brain would have to swell markedly more to cause an increased ICP. ICP can also be negative due to the loss of normal ICP regulation and artificially decreased pulse wave amplitudes [11]. These results may indicate that EVDs and traditional ICP monitoring devices may be less useful, opening the idea of different devices needed to monitor ICP in patients with craniectomies.

The idea of using a durometer for the measurement of intracranial pressure dates back to 1977, when McGraw and Alexander came up with the idea to measure intracranial pressure with a durometer over the skin flap after decompressive craniectomy [12]. They were able to successfully predict intracranial pressure from 5 mmHg to 50 mmHg with a standard error of 2 mmHg [12]. Durometers have also been used for measuring the stiffness of meningiomas to confirm for imaging studies [13], but since 1977, there have been no easily accessible studies on durometers being used as measurement tools to roughly gauge ICP.

A durometer could be used to predict oncoming elevated ICP. This hypothesis comes from the idea that skin turgor would be elevated before ICP would become elevated, as the skin flap would need to be full with brain tissue before elevated pressure begins to increase in the cerebrospinal fluid (CSF) within the brain. To predict a rise in ICP in patients with decompressive craniectomies, measuring skin turgor may be a more effective tool. We sought to correlate durometer readings with intracranial pressure by checking for a rise in skin turgor before a rise in ICP, as well as other measurements that correlate with increased ICP such as optic nerve sheath diameter (ONSD) and Neurological Pupil index (NPi).

## Materials and methods

This study was approved by the Arrowhead Regional Medical Center Institutional Review Board, protocol number 22-41. Patients were included via the following criteria: age >18 and unilateral decompressive craniectomy, with EVD in place. Patients were excluded if they were younger than 18, underwent bilateral decompressive craniectomy, or did not have an ICP monitor. Measurements were collected by a member of the neurosurgical team that had been trained in the collection of each of the data points. To collect data, patients had ICP measured hourly after the EVD was opened, monitoring for a waveform, as regularly practiced. The external ventricular drain was checked regularly by both the team and nursing staff hourly to ensure patency. Skin turgor over the skin flap was measured with a Shore OO durometer (PTC Instruments, Los Angeles, CA) over the center of the defect, avoiding any hard skin, tape, or crust. The skin was only depressed to the depth of the probe of the durometer (<1 mm). This measurement was verified with multiple measurements by a single operator. ONSD was measured with ultrasound with the eye closed and Tegaderm (3M, Saint Paul, MN) covering the eyelid. The optic nerve was measured 3 mm behind the globe, and the diameter of the optic nerve at the widest point was recorded. NPis were recorded with a pupillometer except where there was an eye infection, previous eye surgery, or other confounding events. All of the data was gathered consecutively over the course of months. Statistics were completed using t-test, and correlation coefficients (R) were calculated. A positive R value indicated a positive correlation, while negative indicated a negative correlation; a number closer to one indicated a stronger correlation.

## Results

Fourteen patients were included, with over 100 data points for each ICP, skin turgor, ONSD, and NPi. Each patient had multiple data points collected over several days of admission. None of the patients had multiple data points from the same day. Skin turgor was checked at similar times of the day, except in the instance of elevated ICP seen on the monitor. Five patients went on to have elevated ICP after decompressive hemicraniectomy.

**Figure 1:**
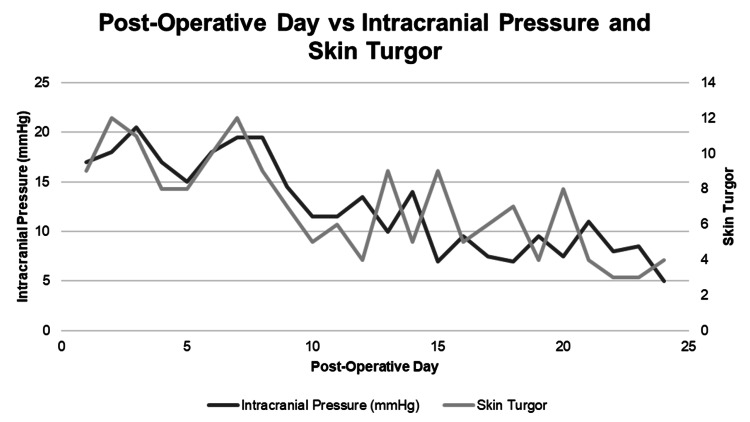
Patient with elevated intracranial pressure (ICP) after decompressive hemicraniectomy.

**Figure 2:**
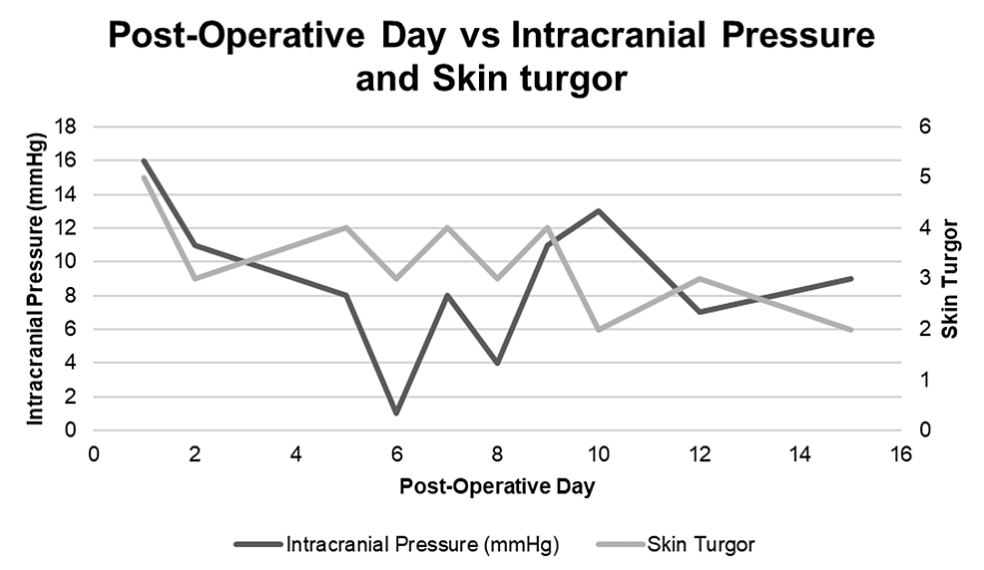
Patient with normal intracranial pressure (ICP) after decompressive hemicraniectomy.

The data shows that a skin turgor of >9 is related to increasing ICP within 24 hours, a skin turgor of 6-9 is a warning, and a skin turgor of <6 is normal. Every patient that showed elevated skin turgor of >9 ended up demonstrating elevated ICP within 24 hours, along with other changes in vital signs that are typical with elevated ICP. None of the patients that consistently had skin turgor of <6 showed evidence of elevated ICP throughout their hospitalization. We show examples of a patient that did not have elevated skin turgor and therefore did not have elevated ICP alongside a patient with elevated skin turgor that had elevated ICP within 24 hours (Figures 1-2).

The R for ONSD to ICP correlation was 0.62. The R for ICP to skin turgor was 0.31. This shows a positive correlation between skin turgor and ICP and ONSD and ICP, with a stronger correlation for ONSD than skin turgor.

## Discussion

As evidenced by the observations of our patients, we see that skin turgor does not correlate directly with ICP as strongly as we have seen with ONSD correlating with ICP. However, elevated skin turgor does result in a later increase in ICP, within 24 hours. ICP monitoring in patients with decompressive hemicraniectomy has been controversial [14]. Several studies have shown that ICP monitors may not reflect rising ICP soon enough, as the craniectomy defect allows for herniation outside of the skull prior to damage to the brain [7,9,11]. This creates a reservoir for the swelling brain to occupy. In addition, as noted earlier, the fluid dynamics of a patient after a decompressive craniectomy could be altered as well [11]. In patients with altered fluid dynamics, ICP readings may not be as accurate. The value and accuracy of an ICP monitor could be skewed due to the lack of a bone flap and altered fluid dynamics. This necessitates the use of adjunct measures to determine the ICP. One such measure includes palpation of the patient’s craniectomy defect site. This is a subjective method to determine ICP, using words such as soft, full, and tense to describe the flap. However, each provider may have a different threshold as to using each term.

An even earlier study demonstrated the correlation between skin turgor using a durometer and corresponding ICP monitor measurements [12]. They showed that elevated ICPs do result in increasing skin turgor that could be measured with the durometer. A durometer could be an objective measure of the subjective palpation that is traditionally used.

No study has developed a temporal relationship between durometer readings and ICP in order to create a predictive method of rising ICPs. By looking at the temporal relationship between skin turgor and ICP, we note a delay between the elevation of the two measurements; the skin turgor increases first followed by an increase in the ICP. By creating this temporal relationship, measures can be taken to decrease the ICP of the patient prior to reaching critical and catastrophic levels. This could be used to prevent further deterioration of the patient’s clinical status.

Limitations of this study include the limited number of patients in the study. In addition, due to the decompressive nature of a hemicraniectomy, most patients did not go on to have elevated ICP throughout their hospital course. In addition, the durometer relies on precision and accuracy, so operator error could result in falsely high or low readings.

## Conclusions

Our data suggests that a durometer could be used as an early warning sign of impending elevated ICP and subsequent herniation. In addition, other metrics, including ICP monitor, vital signs, and clinical exam, should continue to be the mainstay of managing these patients; however, this added tool may provide additional information that could be used to improve patient outcomes. Future studies need to be conducted to further establish the usability and importance of early detection of elevated ICP in hemicraniectomy patients.
